# Univariable associations between a history of incarceration and HIV and HCV prevalence among people who inject drugs across 17 countries in Europe 2006 to 2020 – is the precautionary principle applicable?

**DOI:** 10.2807/1560-7917.ES.2021.26.49.2002093

**Published:** 2021-12-09

**Authors:** Lucas Wiessing, Eleni Kalamara, Jack Stone, Peyman Altan, Luk Van Baelen, Anastasios Fotiou, D’Jamila Garcia, Joao Goulao, Bruno Guarita, Vivian Hope, Marie Jauffret-Roustide, Lina Jurgelaitienė, Martin Kåberg, Adeeba Kamarulzaman, Liis Lemsalu, Anda Kivite-Urtane, Branko Kolarić, Linda Montanari, Magdalena Rosińska, Lavinius Sava, Ilonka Horváth, Thomas Seyler, Vana Sypsa, Anna Tarján, Ioanna Yiasemi, Ruth Zimmermann, Marica Ferri, Kate Dolan, Anneli Uusküla, Peter Vickerman

**Affiliations:** 1 European Monitoring Centre for Drugs and Drug Addiction (EMCDDA), Public Health Unit, Lisbon, Portugal; 2 EASO MTC Block A, Winemakers Wharf, Grand Harbour Valletta, Malta; 3 Population Health Sciences, Bristol Medical School, University of Bristol, Bristol, United Kingdom; 4 Ministry of Health, Public Health General Directorate, Ankara, Turkey; 5 Sciensano, Epidemiology and public health, Lifestyle and chronic diseases, Brussels, Belgium; 6 University Mental Health, Neurosciences, & Precision Medicine Research Institute, Athens, Greece; 7 NOVA FCSH – Universidade Nova de Lisboa, Lisbon, Portugal; 8 General Director on Addictive Behaviours and Dependencies, Ministry of Health, Lisbon, Portugal; 9 Public Health Institute, Liverpool John Moores University, Liverpool, United Kingdom; 10 Centre d’Étude des Mouvements Sociaux (Inserm U1276/CNRS UMR8044/EHESS), Paris, France; 11 Santé Publique France, Saint-Maurice, France; 12 Baldy Center for Law and Social Policy, Buffalo University of Social Sciences, New York, United States; 13 British Columbia Centre on Substance Use (BCCSU), Vancouver, Canada; 14 Drug, Tobacco and Alcohol Control Department, Vilnius, Lithuania; 15 Social Innovations and Science Centre, Vilnius, Lithuania; 16 Stockholm Needle Exchange, Stockholm Centre for Dependency Disorders, Stockholm, Sweden; 17 Department of Medicine Huddinge, Division of Infection and Dermatology, Karolinska Institutet, Karolinska University Hospital Huddinge, Stockholm, Sweden; 18 Department of Medicine, Faculty of Medicine, University of Malaya, Kuala Lumpur, Malaysia; 19 Centre for Prevention of Drug Addiction and Infectious Diseases, National Institute for Health Development, Tallinn, Estonia; 20 Riga Stradins University, Institute of Public Health, Riga, Latvia; 21 Andrija Stampar Teaching Institute of Public Health, Zagreb, Croatia; 22 Medical Faculty, University of Rijeka, Rijeka, Croatia; 23 National Institute of Public Health NIH – National Research Institute, Warsaw, Poland; 24 National Antidrug Agency – Ministry of Internal Affairs, Bucharest, Romania; 25 Gesundheit Österreich GmbH – Austrian National Public Health Institution, International Affairs and Consulting, Vienna, Austria; 26 Department of Hygiene, Epidemiology and Medical Statistics, Medical School, National and Kapodistrian University of Athens, Athens, Greece; 27 Hungarian Reitox National Focal Point, Budapest, Hungary; 28 Monitoring Department, Cyprus National Addictions Authority, Nicosia, Cyprus; 29 Robert Koch Institute, Department of Infectious Disease Epidemiology, Berlin, Germany; 30 Program of International Research and Training, National Drug and Alcohol Research Centre, Sydney, Australia; 31 Department of Family Medicine and Public Health, University of Tartu, Tartu, Estonia

**Keywords:** HIV, HCV, PWID, prison, incarceration, decarceration

## Abstract

**Background:**

People who inject drugs (PWID) are frequently incarcerated, which is associated with multiple negative health outcomes.

**Aim:**

We aimed to estimate the associations between a history of incarceration and prevalence of HIV and HCV infection among PWID in Europe.

**Methods:**

Aggregate data from PWID recruited in drug services (excluding prison services) or elsewhere in the community were reported by 17 of 30 countries (16 per virus) collaborating in a European drug monitoring system (2006–2020; n = 52,368 HIV+/−; n = 47,268 HCV+/−). Country-specific odds ratios (OR) and prevalence ratios (PR) were calculated from country totals of HIV and HCV antibody status and self-reported life-time incarceration history, and pooled using meta-analyses. Country-specific and overall population attributable risk (PAR) were estimated using pooled PR.

**Results:**

Univariable HIV OR ranged between 0.73 and 6.37 (median: 2.1; pooled OR: 1.92; 95% CI: 1.52–2.42). Pooled PR was 1.66 (95% CI 1.38–1.98), giving a PAR of 25.8% (95% CI 16.7–34.0). Univariable anti-HCV OR ranged between 1.06 and 5.04 (median: 2.70; pooled OR: 2.51; 95% CI: 2.17–2.91). Pooled PR was 1.42 (95% CI: 1.28–1.58) and PAR 16.7% (95% CI: 11.8–21.7). Subgroup analyses showed differences in the OR for HCV by geographical region, with lower estimates in southern Europe.

**Conclusion:**

In univariable analysis, a history of incarceration was associated with positive HIV and HCV serostatus among PWID in Europe. Applying the precautionary principle would suggest finding alternatives to incarceration of PWID and strengthening health and social services in prison and after release (‘throughcare’).

## Background

People who inject drugs (PWID) are frequently incarcerated, with an estimated 58% of PWID ever incarcerated and prison populations including up to 50% PWID in many countries [1-3]. Incarceration of PWID is associated with higher risks of drug-related problems [4,5] including human immunodeficiency virus (HIV) and hepatitis C virus (HCV) transmission [6-9], fatal overdose [10,11], mental illness and social disruption [5] and poor or no access to care and treatment [12] – including for women [13] – as well as unfavourable HIV treatment (anti-retroviral treatment (ART)) outcomes [14-16]. However, prisons may also provide an important opportunity for treatment and care [5,12,14,17,18].

Incarceration is associated with increases in HIV- and HCV-related risk behaviour among PWID both in prison and after release [19-22], and may be a driver of HCV and HIV transmission among PWID [6,7,19,23,24]. Incarceration is associated with self-reported transitions to injecting drug use, sharing of injecting equipment inside prison (because of their scarcity), sharing tattooing and shaving materials, and unprotected sex [19,20,25-27]. Injecting and other risks (e.g. sex work, nonfatal overdose and death) have been found to increase immediately after release from prison [21,28-31]. This may be due to the disruption of protective factors and social conditions (e.g. interruption of OST, unemployment), which could be especially marked in countries with repressive policies and insufficient needle and syringe programmes (NSP), opioid substitution treatment (OST), ART and HCV treatment and other services [23,28,32,33].

While a minority of countries (many of these in Europe) have expanded services such as OST, NSP and ART/HCV treatment in the community, services in prisons are still mostly lacking [34-37]. There is limited access to OST for incarcerated PWID, an almost complete absence of NSP and a lack of options for stimulant users, in prison and/or after release [36,38]. ‘Throughcare’, the uninterrupted provision of services to an individual from community to prison and back to community after release, is seldom in place [33,39,40].

Law enforcement and incarceration may not be effective in reducing drug use, while resulting in high public health and social costs [41,42]. Abolishing incarceration for use and possession of illicit drugs can result in a major reduction of incarceration episodes and in important health improvements among people who use drugs [6,42-47]. For example, decriminalisation of minor drug offences in Portugal has resulted in important reductions in the number of individuals incarcerated for drug law offences, significant savings in legal system costs, large public health benefits and declines in problem drug use (i.e. a shift from heroin use to cannabis use) [43,46,48,49].

To assess the degree to which a history of incarceration may be associated with a positive HIV and HCV antibody status among PWID in countries in Europe, we used data from the European Monitoring Centre for Drugs and Drug Addiction (EMCDDA) drug monitoring system. Using the population attributable risk (PAR), we estimated the proportion of HIV and HCV seropositivity among PWID that can be attributed to a history of incarceration if these associations are causal. This gives an estimate of the degree to which HIV and HCV transmission among PWID could potentially have been avoided through decarceration-oriented drug policies.

## Methods

### Data sources

The EMCDDA monitoring system receives aggregate data on HIV and HCV prevalence in PWID annually from collaborating countries. For the time period reported here (2006 to 2020), this system covered 28 countries of the European Union (EU) plus Turkey and Norway, with 17 of the 30 countries (16 per virus) being able to provide the data required for this analysis. Fifteen countries provided data for both viruses, Belgium only for HIV and Turkey only for HCV (the United Kingdom (UK) provided data for 2006 to 2015, when it was an EU Member State). The data for this analysis include information on HIV and HCV antibody test result and a self-reported history of incarceration, except for all HIV data from Belgium and part of the HIV data from Latvia, which are based on self-reported test status. PWID include ever and recent injectors recruited at drug services (excluding prison services) or elsewhere in the community. For more information on data sources, see the Supplement (primary study characteristics in Tables S1 and S2 and references) and https://www.emcdda.europa.eu/data/stats2021/drid_en.

Aggregate HIV and HCV testing data (not individual case records) were available for analysis as two separate datasets, one for HIV and one for HCV (thus totals tested are different per virus). Each dataset contained only total counts by country (number tested, number and percentage seropositive and -negative for HIV or HCV) broken down by self-reported prison history (ever/never in prison). In addition, we obtained study-level data on recruitment method and setting.

### Statistical analyses

Univariable country-level odds ratios (OR), prevalence ratios (PR – analogous to a relative risk [50]) and PAR and their 95% confidence intervals (CI) were calculated between a history of incarceration and HIV and HCV infection. The OR and PR were pooled using random effects meta-analysis (DerSimonian-Laird methodology), with a continuity correction of 0.5 applied where countries contained a zero cell. We evaluated between-study heterogeneity using the I^2^ statistic and the p value for heterogeneity (Cochran’s *Q* statistic). The proportion of PWID ever incarcerated was used with the pooled PR to estimate a ‘pooled PAR’. We calculated the correlation coefficients between the country-specific OR for HIV and HCV and between the country-specific PAR for HIV and HCV, to assess consistency in the associations between each of these infections and a history of incarceration (Pearson’s r, using the CORREL function in MS Excel).

We carried out a sensitivity analysis to examine the extent to which the pooled OR was affected by differences in the prevalence of: (i) history of incarceration, (ii) HIV or (iii) HCV in the PWID population, (iv) recruitment method, (v) recruitment setting, (vi) gross domestic product (GDP) per capita, (vii) national incarceration rate and (viii) region (as defined by the United Nations [51]). We grouped countries as follows: (i) percentage of PWID reporting a history of incarceration: < 45% = low, 45–55% = medium and > 55% = high, (ii) HIV prevalence: < 5% = low, 5–20% = medium and > 20% = high, (iii) HCV prevalence: < 45% = low, 45–65% = medium and > 65% = high, (iv) recruitment method: ‘seroprevalence studies’ (in which an unbiased estimate of prevalence is attempted) vs ‘diagnostic testing’ studies (that use test results from routine testing in services), (v) recruitment setting ‘exclusively low-threshold services such as NSP, and/or on the street’, ‘both low-threshold services/street and drug treatment settings’ and ‘exclusively drug treatment settings’, (vi) GDP per capita (World bank data), using the median across all countries in our analysis: < USD 20,000 (the equivalent of EUR 17,478 on 15 November 2021) = low and ≥ USD 20,000 = high, (vii) national incarceration rate [52], using the median across all countries: < 107 per 100,000 = low and ≥ 107 per 100,000 = high, and (viii) United Nations region: northern Europe, eastern Europe, western Europe and southern Europe (with Cyprus and Turkey classified as southern Europe).

A chi-squared test for heterogeneity across subgroup estimates was performed to test for statistical differences between the groups. Analyses were done in MS Excel (v. 14.5.7) and Stata (StataCorp. 2019. Stata Statistical Software: Release 16. StataCorp LLC, College Station, US).

## Results

The total number tested for HIV was 52,368 and for HCV 47,268. Sample sizes for HIV were large in most countries, with a median of 916 (interquartile range (IQR): 528–1,943) and a mean of 3,273, although they varied considerably, from 181 PWID tested in Poland to 29,061 in the UK (Table 1 and Supplementary Table S1). Of those tested for HIV, 62.1% (n = 32,524) had ever been in prison, of whom 5.7% (95% CI: 5.5–6.0; n = 1,861) were HIV-positive. Among those ever in prison, HIV prevalence also varied greatly across countries (0–64%), with a pooled average of 5.7% (median: 6.2%; IQR: 1.0–23.6). HIV prevalence among those with no history of incarceration (37.9% of all those tested for HIV; n = 19,844) had a pooled average of 5.0% (95% CI: 4.7–5.3; n = 992), with similarly wide variation across countries (range: 0–48; median: 2.4%; IQR: 1.1–12.0).

**Table 1:** HIV prevalence by self-reported past incarceration among people who inject drugs in 16 European countries, 2006–2020 (n = 52,368)

Country	Year	n	Ever in prison				Never in prison		
			n	%	Positive	% positive	n	Positive	% positive
Cyprus	2006–15	888	362	40.8	1	0.3	526	2	0.4
United Kingdom	2006–15	29,061	20,323	69.9	234	1.2	8,738	114	1.3
Austria	2006–15	608	220	36.2	1	0.5	388	2	0.5
Croatia	2007	397	167	42.1	0	0.0	230	0	0.0
Portugal	2010–15	1,901	966	50.8	216	22.4	935	149	15.9
Romania	2015	522	199	38.1	71	35.7	323	80	24.8
Latvia	2007–14	3,047	1,552	50.9	424	27.3	1,495	256	17.1
Estonia	2012–14	1,277	739	57.9	473	64.0	538	256	47.6
France	2011	898	542	60.4	92	17.0	356	30	8.4
Germany	2011–14	2,069	1,672	80.8	91	5.4	397	10	2.5
Sweden	2013–20	8,512	4,326	50.8	154	3.6	4,186	60	1.4
Greece	2006–15	934	164	17.6	11	6.7	770	18	2.3
Poland	2009	181	88	48.6	24	27.3	93	10	10.8
Belgium**^a^**	2008–11	363	210	57.9	12	5.7	153	2	1.3
Lithuania	2012–14	530	420	79.2	54	12.9	110	3	2.7
Hungary	2014–15	1,180	574	48.6	3	0.5	606	0	0.0
**Total^b^**		**52,368**	**32,524**	**62.1**	**1,861**	**5.7**	**19,844**	**992**	**5.0**
**Median (unweighted)^c^**		**916**	**481**	**50.8**	**63**	**6.2**	**462**	**14**	**2.4**

EMCDDA: European Monitoring Centre for Drugs and Drug Addiction; PWID: people who inject drugs.

Countries are listed in the order of increasing odds ratio. For sample characteristics see Supplementary Table S1. Given varying recruitment methods and settings and geographical coverage, the data should not be interpreted as being representative for all PWID in the country, only for the sample studied. Data for Belgium and part of the data for Latvia concern self-reported HIV status. Data for Hungary were not controlled for double counting between the two study years and may include duplicates. Data for Sweden were obtained directly from the Swedish author, not through the EMCDDA monitoring system.

^a^ In Belgium, data were from the French-speaking community only.

**^b^** Pooled percentages are the weighted average.

^c^ Unweighted medians are not necessarily consistent with one another as they merely reflect the midpoint of the 16 country values in that column (unlike the pooled data that all describe the same sample of n = 52,368 each median only describes one column with 16 country level values, thus providing an alternative unweighted central value. For the percentages these can be interpreted as if all countries had the same sample size).

For HCV, total sample sizes were somewhat smaller than those reported for HIV (mostly provided by the same studies, see Supplement), with a median of n = 641 (IQR: 393.5–1,222.5) and a mean of 2,954, ranging from 168 PWID tested in Turkey to 28,536 in the UK (Table 2 and Supplementary Table S2). Of those tested for HCV, 62.7% (n = 29,633) had ever been in prison, of whom 59.0% (95% CI: 58.5–59.6; n = 17,493) were HCV antibody-positive. The HCV antibody prevalence among those with no history of incarceration (37.3% of all those tested for HCV; n = 17,635) was 44.0% (95% CI: 43.3–44.7; n = 7,760). HCV prevalence among those ever in prison varied greatly across countries (8–92%) with the median at 69.3% (IQR: 58.8–84.9). HCV prevalence among those never in prison also varied widely (5–84%), with a median of 47.1% (IQR: 37.0–60.5).

**Table 2:** HCV antibody prevalence by self-reported past incarceration among people who inject drugs in 16 European countries, 2006–2020 (n = 47,268)

Country	Year	n	Ever in prison				Never in prison		
			n	%	Positive	% positive	n	Positive	% positive
Portugal	2010–15	1,518	762	50.2	648	85.0	756	637	84.3
Turkey	2008	168	25	14.9	2	8.0	143	7	4.9
Cyprus	2006–15	894	363	40.6	193	53.2	531	206	38.8
United Kingdom	2006–15	28,536	19,914	69.8	10,164	51.0	8,622	2,800	32.5
Poland	2009	180	87	48.3	61	70.1	93	47	50.5
Croatia	2007	397	167	42.1	99	59.3	230	87	37.8
Germany	2011–14	2,071	1,674	80.8	1,131	67.6	397	178	44.8
Austria	2006–15	608	220	36.2	126	57.3	388	129	33.2
Greece	2006–15	583	108	18.5	74	68.5	475	212	44.6
Sweden	2013–20	8,512	4,326	50.8	3,453	79.8	4,186	2,485	59.4
Latvia	2014	383	164	42.8	150	91.5	219	174	79.5
Romania	2015	521	199	38.2	172	86.4	322	223	69.3
France	2011	898	542	60.4	402	74.2	356	176	49.4
Lithuania	2014	200	125	62.5	106	84.8	75	48	64.0
Hungary	2014–15	1,124	548	48.8	356	65.0	576	199	34.5
Estonia	2013–14	675	409	60.6	356	87.0	266	152	57.1
**Total^a^**		**47,268**	**29,633**	**62.7**	**17,493**	**59.0**	**17,635**	**7,760**	**44.0**
**Median (unweighted)^b^**		**641**	**292**	**48.5**	**183**	**69.3**	**372**	**177**	**47.1**

EMCDDA: European Monitoring Centre for Drugs and Drug Addiction; PWID: people who inject drugs.

Countries are listed in the order of increasing odds ratio. For sample characteristics see Supplementary Table S2. Given varying recruitment methods and settings and geographical coverage, the data should not be interpreted as being representative for all PWID in the country, only for the sample studied. Data for Hungary were not controlled for double counting between the two study years and may include duplicates. Data for Sweden were obtained directly from the Swedish author, not through the EMCDDA monitoring system.

^a^ Pooled percentages are the weighted average.

^b^ Unweighted medians are not necessarily consistent with one another as they merely reflect the midpoint of the 16 country values in that column (unlike the pooled data that all describe the same sample of n = 47,268 each median only describes one column with 16 country level values, thus providing an alternative unweighted central value. For the percentages these can be interpreted as if all countries had the same sample size).

Levels of self-reported history of incarceration (‘ever in prison’) also diverged across countries. In the HIV testing dataset, having ever been in prison ranged from 17.6% in Greece to 80.8% in Germany, with a pooled average across countries of 62.1% (unweighted median: 50.8%; IQR: 41.7–58.5). A similar pattern was observed in the HCV testing dataset (ranging from 14.9% in Turkey to 80.8% in Germany) with a pooled average of 62.7% (unweighted median: 48.5%; IQR: 40.0–60.4) (Tables 1 and 2).

### Association between HIV or HCV serostatus and history of incarceration

**Figure 1:**
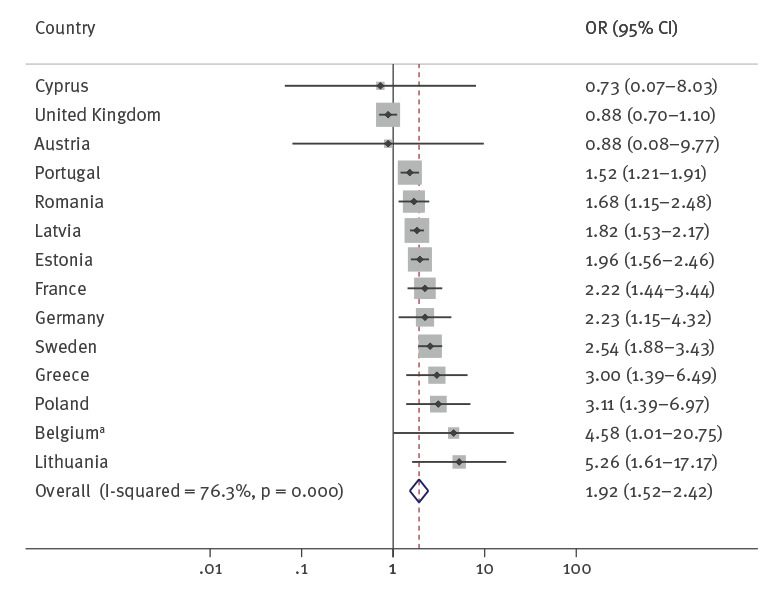
Odds ratios of HIV infection among PWID reporting a history of incarceration vs PWID not reporting a history of incarceration in Europe, 2006–2020 (n = 52,368)

CI: confidence interval; OR: odds ratios; PWID: people who inject drugs.

The OR between a positive HIV serostatus and a history of incarceration showed strong and consistent univariable associations in most countries although with important variation across countries (from 0.73 in Cyprus to 6.37 in Hungary – although these extremes were both not statistically significant) and in some cases wide CI. Eleven of the 16 countries with available data showed a positive association that was statistically significant, while none showed a statistically significant negative association (in five countries, the OR was not statistically significant: two above and three below OR = 1.00). The pooled OR across countries showed a statistically significant positive association (OR = 1.92; 95% CI: 1.52–2.42) (Figure 1 and Table 3) with substantial heterogeneity between countries (I^2^ = 76.3%; p = 0.000).

Data from Belgium and part of the data for Latvia concern self-reported HIV status by the PWID.

**^a^** In Belgium, data were from the French-speaking community only.

**Table 3:** Associations between HIV prevalence and self-reported past incarceration among people who inject drugs in 16 European countries, 2006–2020 (n = 52,368)

Country	Year	OR	95% CI	PR	95% CI	PAR (%)	95% CI
Cyprus	2006–15	0.73^a^	0.07–8.03	0.73^a^	0.07–7.98	NA**^a^**	
United Kingdom	2006–15	0.88^a^	0.70–1.10	0.88^a^	0.71–1.10	NA**^a^**	
Austria	2006–15	0.88^a^	0.08–9.77	0.88^a^	0.08–9.67	NA**^a^**	
Croatia	2007	(1.38)^a^	(0.03–69.9)	NA**^a^**		NA**^a^**	
Portugal	2010–15	1.52	1.21–1.91	1.40	1.16–1.69	16.9	7.52–26.0
Romania	2015	1.68	1.15–2.48	1.44	1.10–1.88	14.4	3.67–25.1
Latvia	2007–14	1.82	1.53–2.17	1.60	1.39–1.83	23.4	16.6–29.7
Estonia	2012–14	1.96	1.56–2.46	1.35	1.21–1.49	16.8	10.8–22.1
France	2011	2.22	1.44–3.44	2.01	1.36–2.97	37.9	17.8–54.3
Germany	2011–14	2.23	1.15–4.32	2.16	1.14–4.11	48.4	10.2–71.5
Sweden	2013–20	2.54	1.88–3.43	2.48	1.85–3.34	42.9	30.2–54.3
Greece	2006–15	3.00	1.39–6.49	2.87	1.38–5.96	24.7	6.26–46.6
Poland	2009	3.11	1.39–6.97	2.54	1.29–4.99	42.8	12.4–66.0
Belgium**^b^**	2008–11	4.58	1.01–20.8	4.37^a^	0.99–19.3	NA**^a^**	
Lithuania	2012–14	5.26	1.61–17.2	4.71	1.50–14.8	74.6	28.4–91.6
Hungary	2014–15	(6.37)^a^	(0.32–127)	NA**^a^**		NA**^a^**	
**Total (pooled)^c^**		**1.92**	**1.52–2.42**	**1.66**	**1.38**–**1.98**	**25.8**	**16.7**–**34.0**
**Median (unweighted)**		**2.09**	**NA**	**1.81**	**NA**	**37.9**	**NA**

CI: confidence interval; EMCDDA: European Monitoring Centre for Drugs and Drug Addiction; NA: not applicable; OR: odds ratio; PAR: population attributable risk; PR: prevalence ratio (necessary to calculate PAR); PWID: people who inject drugs.

^a^ Not statistically significant (two-sided p > 0.05). NA: Country-specific PAR was not calculated where PR was not statistically significant or NA. Country-specific OR shown in brackets, PR not calculated and countries excluded from the pooled totals where OR was estimated with adjustment for an empty cell (Croatia, Hungary).

^b^ In Belgium, data were from the French-speaking community only.

^c^ OR and PR pooled using random effects meta-analysis to account for heterogeneity between countries, excluding Croatia and Hungary. Data for Belgium and part of the data for Latvia concern self-reported HIV status. Data for Hungary were not controlled for double counting between the two study years and may include duplicates. Data for Sweden were obtained directly from the Swedish author, not through the EMCDDA monitoring system.

Countries are listed in order of increasing odds ratio. For sample characteristics see Supplementary Table S1. Given varying recruitment methods and settings and geographical coverage, the data should not be interpreted as being representative for all PWID in the country, only for the sample studied.

**Figure 2:**
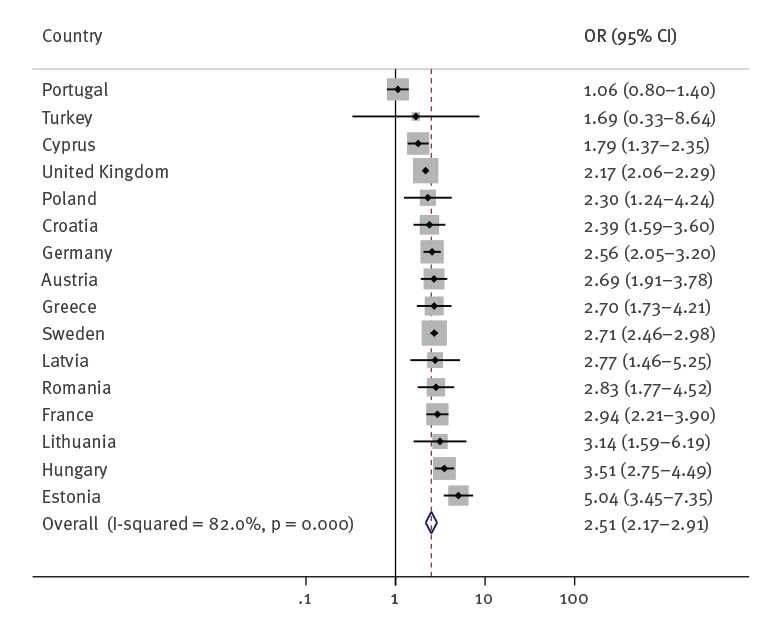
Odds ratio of HCV antibodies among PWID reporting a history of incarceration vs PWID not reporting a history of incarceration in Europe, 2006–2020 (n = 47,268)

CI: confidence interval; OR: odds ratios; PWID: people who inject drugs.

The OR for a positive HCV serostatus and a history of incarceration also showed strong and consistent univariable associations in most countries although, similarly to HIV, with wide variation between countries (from 1.06 in Portugal to 5.04 in Estonia – although the estimate for Portugal was not statistically significant) and in some cases wide CI. Fourteen of the 16 countries with available data showed a statistically significant positive association, two showed a non-significant positive association and none showed a negative association. The pooled OR across countries was large, suggesting more than a doubling in the odds of HCV antibody positivity among PWID who were ever in prison compared with those never in prison (OR = 2.51; 95% CI: 2.17–2.91) (Figure 2 and Table 4). As for HIV, there was substantial heterogeneity between countries (I^2^ = 82.0%, p = 0.000).

**Table 4:** Associations between HCV antibody prevalence and self-reported past incarceration among people who inject drugs in 16 European countries, 2006–2020 (n = 47,268)

Country	Year	OR	95% CI	PR	95% CI	PAR (%)	95% CI
Portugal	2010–15	1.06^a^	0.80–1.40	1.01	0.97–1.05	NA^a^	
Turkey	2008	1.69^a^	0.33–8.64	1.63	0.36–7.42	NA^a^	
Cyprus	2006–15	1.79	1.37–2.35	1.37	1.19–1.58	13.1	7.16–19.1
United Kingdom	2006–15	2.17	2.06–2.29	1.57	1.52–1.61	28.5	26.6–29.9
Poland	2009	2.30	1.24–4.24	1.39	1.09–1.77	15.9	4.17–27.1
Croatia	2007	2.39	1.59–3.60	1.57	1.27–1.93	19.3	10.2–28.1
Germany	2011–14	2.56	2.05–3.20	1.51	1.34–1.69	29.2	21.6–35.8
Austria	2006–15	2.69	1.91–3.78	1.72	1.44–2.07	20.7	13.7–27.9
Greece	2006–15	2.70	1.73–4.21	1.54	1.31–1.81	9.09	5.43–13.0
Sweden	2013–20	2.71	2.46–2.98	1.34	1.31–1.38	14.9	13.6–16.2
Latvia	2014	2.77	1.46–5.25	1.15	1.06–1.25	6.04	2.50–9.67
Romania	2015	2.83	1.77–4.52	1.25	1.14–1.37	8.72	5.08–12.4
France	2011	2.94	2.21–3.90	1.50	1.34–1.69	23.2	17.0–29.4
Lithuania	2014	3.14	1.59–6.19	1.33	1.10–1.59	17.1	5.88–26.9
Hungary	2014–15	3.51	2.75–4.49	1.88	1.65–2.14	30.0	24.1–35.7
Estonia	2013–14	5.04	3.45–7.35	1.52	1.36–1.70	24.0	17.9–29.8
**Total (pooled)** ^b^		**2.51**	**2.17**–**2.91**	**1.42**	**1.28**–**1.58**	**16.7**	**11.8**–**21.7**
**Median (unweighted)**		**2.70**	**NA**	**1.51**	**NA**	**18.2**	**NA**

CI: confidence interval; EMCDDA: European Monitoring Centre for Drugs and Drug Addiction; NA: not applicable; OR: odds ratio; PAR: population attributable risk; PR: prevalence ratio (necessary to calculate PAR); PWID: people who inject drugs.

^a^ Not statistically significant (two-sided p > 0.05). NA: Country-specific PAR was not calculated where PR was not statistically significant.

^b^ OR and PR pooled using random effects meta-analysis to account for heterogeneity between countries. Data for Hungary were not controlled for double counting between the two study years and may include duplicates. Data for Sweden were obtained directly from the Swedish author, not through the EMCDDA monitoring system.

Countries are listed in order of increasing odds ratio. For sample characteristics see Supplementary Table S2. Given varying recruitment methods and settings and geographical coverage, the data should not be interpreted as being representative for all PWID in the country, only for the sample studied.

### Population attributable risk of HIV or HCV seropositivity

The estimated PAR of HIV seropositivity, that is the fraction of positive cases attributable to a history of incarceration if the association are causal, varied from 14.4% in Romania to 74.6% in Lithuania, with an overall pooled PAR of 25.8% (95% CI: 16.7–34.0) (Table 3). For HCV, it ranged from 6.0% in Latvia to 30.0% in Hungary, with an overall pooled PAR of 16.7% (95% CI: 11.8–21.7) (Table 4).

The correlation coefficient between the OR for HIV and HCV was low (r = 0.3, based on the 15 countries with information on both viruses) as was the correlation coefficient between the PAR for HIV and HCV (r = 0.35, based on nine countries with PAR available for both viruses) (not shown in Tables), suggesting differences between the level of HIV and HCV transmission associated with a history of incarceration. In most countries, the OR was higher for HCV than for HIV (10 of 15 countries), while the PAR was mostly lower for HCV than for HIV (eight of nine countries).

### Sensitivity analyses

In sensitivity analyses, the OR for HCV differed significantly based on geographical region, with pooled estimates greatest in northern (OR = 2.85; 95% CI: 2.26–3.59), eastern (OR = 3.21; 95% CI: 2.62–3.95) and western Europe (OR = 2.70; 95% CI: 2.31–3.15) and lowest in southern Europe (OR = 1.82; 95% CI: 1.23–2.68) (Table 5). A similar but weaker pattern was found for HIV – with the pooled OR not significantly different. No other statistical differences were detected, owing to low statistical power at country level (for these sub-analyses, the sample size is 16 countries per virus).

**Table 5:** Pooled odds ratios from sensitivity analyses, by study characteristics, HIV (n = 51,627 tests^a^) and HCV (n = 47,268 tests) among people who inject drugs related to past incarceration, 16 European countries, 2006–2020

	HIV		p value for difference	HCV		p value for difference
	OR	95% CI		OR	95% CI	
HIV/HCV prevalence						
Low (< 5%)	1.89	1.02–3.49	0.743	2.13	1.54–2.96	0.513
Medium (5–20%)	2.22	1.49–3.31		2.62	2.20–3.12	
High (> 20%)	1.85	1.62–2.10		2.61	1.69–4.05	
Incarceration prevalence						
Low (< 45%)	1.83	1.30–2.56	0.933	2.31	1.98–2.71	0.386
Medium (45–55%)	1.96	1.53–2.50		2.2	1.37–3.55	
High (> 55%)	2.02	1.22–3.33		2.91	2.21–3.82	
Recruitment method						
Seroprevalence studies	1.86	1.37–2.53	0.656	2.86	2.36–3.46	0.084
Diagnostic studies	2.09	1.42–3.08		2.04	1.42–2.92	
Recruitment setting^b^						
Exclusively in LTS	2.04	1.75–2.39	0.635	2.89	2.45–3.41	0.143
LTS and DTS	1.37	0.55–3.40		2.69	2.05–3.52	
Exclusively in DTS	1.86	1.19–2.93		1.9	1.22–2.94	
GDP per capita^c^						
Low (<USD 20,000)	2.09	1.58–2.75	0.442	2.96	2.52–3.47	0.077
High (>USD 20,000)	1.76	1.26–2.47		2.37	1.96–2.86	
National incarceration rate						
Low (< 107/100,000)	2.19	1.80–2.66	0.308	2.55	2.26–3.59	0.981
High (≥ 107/100,000)	1.81	1.32–2.50		2.54	1.87–3.45	
Region						
Eastern Europe	2.06	1.17–3.62	0.911	3.21	2.62–3.95	0.044
Northern Europe	2.09	1.70–2.58		2.85	2.26–3.59	
Southern Europe	1.79	1.06–3.00		1.82	1.23–2.68	
Western Europe	1.73	0.90–3.33		2.70	2.31–3.15	

CI: confidence interval; DTS: drug treatment services/settings; GDP: gross domestic product; HCV: hepatitis C virus; LTS: low threshold setting; OR: odds ratio.

^a^ Croatia and Hungary were excluded from the HIV analysis for consistency with Table 3 (instability of the OR due to zero /low number of HIV cases), therefore HIV sample size was reduced by n = 741.

^b^ LTS such as needle and syringe programmes, street recruitment.

^c^ World Bank data, approximate value is EUR 17,478 using conversion rate per 15 November 2021.

p value for chi-squared test for heterogeneity between sub-groups.

## Discussion

Our findings suggest that past incarceration is associated in univariable analysis with a positive HIV and HCV serostatus among PWID in Europe. We estimate that the PAR, i.e. the percentage of infections attributable to a history of incarceration if these associations are causal, would be around one in four infections (25.8%) for HIV and one in six (16.7%) for HCV.

The country-specific PAR ranged from 14.4% in Romania to 74.6% in Lithuania for HIV and from 6.0% in Latvia to 30.0% in Hungary for HCV. This seems to align well with other epidemiological evidence. In Lithuania, a large HIV outbreak in prisons has occurred [53], and new HIV diagnoses in prisons were increasing until 2017 [54]. In Estonia (with a PAR for HCV of 24.0%) high prevalence of HCV and HIV were found in prisons, and very low HCV treatment rates, with HCV seropositivity strongly associated with a history of drug use, HIV co-infection, previous incarceration and increasing age [55]. Conversely, the weak association for HIV in Romania (with the lowest PAR at 14.4%), may reflect high transmission unrelated to incarceration, possibly a consequence of a recent HIV outbreak among PWID in the community and high heterosexual transmission in the general population following an earlier large nosocomial HIV outbreak [56]. Portugal showed one of the weakest associations for HIV (and one of the lowest PAR at 16.9%), and no association for HCV, more than a decade after decriminalising minor drug offences (in 2001) and introducing widespread harm reduction measures (since around 1996). Similarly, the UK, which historically has had strong harm reduction policies (although more recently it has lagged behind some other European countries e.g. in introduction of drug consumption rooms), showed no association for HIV and one of the weakest for HCV. However, it should be noted that the data we used were from England and Wales, where HIV prevalence is very low, whereas a very strong association between HIV and a history of incarceration has been found during a recent HIV outbreak among PWID in Scotland [57]. By contrast, Sweden, a country with a traditionally more repressive drug policy and low levels of harm reduction (although with a major increase in NSP and OST in the last decade), showed strong associations both for HIV and HCV.

The strength of most of the (positive) associations we found between a history of incarceration and a positive HIV or HCV serostatus, and the fact that we observed these associations across a large number of countries, should strengthen confidence in our results. Moreover, no country showed a statistically significant negative association, and the associations were seen across studies using different recruitment methods and for two blood-borne viruses.

However, our study has important limitations. Notably, using aggregate data, we were unable to adjust for potential individual-level confounders such as the number of years injecting or age, which have been shown to be strongly related to HIV and HCV infection as well as a history of incarceration, thus potentially resulting in overestimated or even spurious associations [58]. Further, we do not have information on when infection occurred, before, during or after incarceration, and we are unable to analyse the effects of the number of times incarcerated or total time spent in prison. Therefore, our findings remain inconclusive, and need to be interpreted in the context of a wealth of similar findings from other studies [7,33,57,59-69].

Individual level variables may play a further role in the causal pathway between incarceration and HIV/HCV infection, such as having ever shared needles/syringes or number of lifetime sexual partners (e.g. through sex work because of temporary homelessness) or tattooing, and structural variables including national drug policies (existence of sustainable harm reduction policy, access to ART and HCV treatment for PWID, law enforcement regarding drug use etc). Therefore, multivariate adjustment for injecting risks such as injecting frequency and sharing needles/syringes may also result in biased findings, such as underestimating or obscuring a true association between a history of incarceration and HIV/HCV prevalence, if these injecting risks are part of the causal pathway [19-22,33]. Moreover, although different confounders and effect modifiers (e.g. national drug and incarceration policies) are likely to result in different effect sizes in different countries and studies (as we find in our data), the general consistency in our results and with other studies makes it seem unlikely that the causal mechanism for these associations would be fundamentally different, i.e. driven by different factors, between countries and studies. This provides further support for our findings, despite their limitations, and with special relevance for the countries here studied.

We are less confident about some of the lowest levels of incarceration history among PWID in our data and suspect it may have been under-reported in some countries (possibly because it is highly stigmatised), especially where these seem inconsistent with overall population incarceration rates [70]. However, misclassification of incarceration history because of under-reporting would lead to underestimating the strength of the associations with HIV or HCV, the PR and PAR, thus strengthening the validity of our findings. Some of our data are not recent (since 2006) or based on only one calendar year, while since 2007, there has been much effort with respect to bringing down incidences of HCV and HIV and there have been downward trends in the general population and among PWID. However, reductions in prevalence would not necessarily affect the associations here reported, while in one country, we were able to verify that the strong associations in a recent (2013–2020) and large dataset here reported already existed in a smaller independent dataset from 2007 (not shown).

PWID reported life-time prison experience, thus our findings may relate to incarcerations that took place well before the study years. However, we have little indication that incarceration polices for PWID (and services in prisons) have substantially changed across most countries, with the few exceptions here discussed (e.g. Portugal decriminalised in 2001, so that effects of that change should be reflected in our data). Data collection methods and sample representativity varied across countries and findings can probably not be generalised to all PWID at the national level, however, our findings are consistent despite this limitation. Finally, our analysis is cross-sectional so that we are unable to establish the direction (temporality) of potential causality, and while it may be more likely that incarceration leads to HIV and HCV infections rather than the other way round, it is possible that high-risk PWID may be more likely to get incarcerated and to acquire HIV or HCV, thus potentially resulting in selection bias and confounding [71].

Given their consistency across countries and with other studies, our findings may have implications for health and drug policies regarding incarceration of PWID and HIV and HCV infection, even without considering other outcomes such as overdose and mortality. They add further evidence for strengthening service provision throughout incarceration and on release (‘throughcare’) and for considering alternatives to incarceration such as decarceration and decriminalisation (depenalisation, police diversion) policies, which have already been successfully introduced [43,48,49,72,73]. The COVID-19 pandemic has provided additional urgency to reducing the potential negative public health impact resulting from incarcerating people who use drugs, and decarceration has already been applied as a precaution to limit the spread of COVID-19 [74-76].

Despite the limitations with respect to analyses performed and the data available, the precautionary principle states that in a situation of incomplete evidence, it is important to weigh potential costs of inaction (here: continued infections and other harms that are potentially due to incarceration) against costs of action (here: the costs of strengthening services and/or introducing alternatives to incarceration) [77,78]. Thus, (i) if the precautionary principle is applicable to this policy area and (ii) given the potential on-going health costs if our findings and similar findings from other studies do reflect true associations through a common causal process, it would be important to review public health harms among PWID related to incarceration policies even before final conclusive evidence may become available. This would need to address mechanisms to avoid the incarceration of PWID, as well as strengthening services in prisons and after release [43]. If our findings are not confirmed in future work, the established benefits of such service provision and alternatives to incarceration on the health and well-being of PWID, and their cost-effectiveness, are still likely to result in important health improvements among PWID [43,79].

## Conclusion

A history of incarceration was found in univariable analysis to be associated with an increased risk of HIV and HCV seropositivity among community-recruited PWID in Europe. Owing to study limitations these findings should be interpreted with caution. However, our findings are in agreement with other evidence and suggest a need for further in-depth studies. If the precautionary principle is applied, they already suggest a need for reviewing incarceration policies affecting PWID and strengthening health services for PWID, both in prison and after release (‘throughcare’).

## Supplementary Data

120000 Supplement

## References

[1] Dolan K, Khoei EM, Brentari C, Stevens A. Prisons and Drugs: A global review of incarceration, drug use and drug services. Oxford: The Beckley Foundation; 2007. Available from: https://www.beckleyfoundation.org/wp-content/uploads/2016/04/BF_Report_12.pdf

[2] World Health Organization Regional Office for Europe (WHO/Europe). Prisons and health. Copenhagen: WHO/Europe; 2014. Available from: http://www.euro.who.int/__data/assets/pdf_file/0005/249188/Prisons-and-Health.pdf?ua=1

[3] Degenhardt L Peacock A Colledge S Leung J Grebely J Vickerman P Global prevalence of injecting drug use and sociodemographic characteristics and prevalence of HIV, HBV, and HCV in people who inject drugs: a multistage systematic review. Lancet Glob Health. 2017;5(12):e1192-207. 10.1016/S2214-109X(17)30375-3 29074409 PMC5683738

[4] Wood E Werb D Kazatchkine M Kerr T Hankins C Gorna R Vienna Declaration: a call for evidence-based drug policies. Lancet. 2010;376(9738):310-2. 10.1016/S0140-6736(10)60958-0 20650517

[5] Sugarman OK Bachhuber MA Wennerstrom A Bruno T Springgate BF . Interventions for incarcerated adults with opioid use disorder in the United States: A systematic review with a focus on social determinants of health. PLoS One. 2020;15(1):e0227968. 10.1371/journal.pone.0227968 31961908 PMC6974320

[6] Dolan K Wirtz AL Moazen B Ndeffo-Mbah M Galvani A Kinner SA Global burden of HIV, viral hepatitis, and tuberculosis in prisoners and detainees. Lancet. 2016;388(10049):1089-102. 10.1016/S0140-6736(16)30466-4 27427453

[7] Stone J Fraser H Lim AG Walker JG Ward Z MacGregor L Incarceration history and risk of HIV and hepatitis C virus acquisition among people who inject drugs: a systematic review and meta-analysis. Lancet Infect Dis. 2018;18(12):1397-409. 10.1016/S1473-3099(18)30469-9 30385157 PMC6280039

[8] Larney S Kopinski H Beckwith CG Zaller ND Jarlais DD Hagan H Incidence and prevalence of hepatitis C in prisons and other closed settings: results of a systematic review and meta-analysis. Hepatology. 2013;58(4):1215-24. 10.1002/hep.26387 23504650 PMC3723697

[9] Wirtz AL Yeh PT Flath NL Beyrer C Dolan K . HIV and viral hepatitis among imprisoned key populations. Epidemiol Rev. 2018;40(1):12-26. 10.1093/epirev/mxy003 29688317 PMC5982732

[10] Zlodre J Fazel S . All-cause and external mortality in released prisoners: systematic review and meta-analysis. Am J Public Health. 2012;102(12):e67-75. 10.2105/AJPH.2012.300764 23078476 PMC3519300

[11] Merrall EL Kariminia A Binswanger IA Hobbs MS Farrell M Marsden J Meta-analysis of drug-related deaths soon after release from prison. Addiction. 2010;105(9):1545-54. 10.1111/j.1360-0443.2010.02990.x 20579009 PMC2955973

[12] Degenhardt L Larney S Kimber J Gisev N Farrell M Dobbins T The impact of opioid substitution therapy on mortality post-release from prison: retrospective data linkage study. Addiction. 2014;109(8):1306-17. 10.1111/add.12536 24612249

[13] Strathdee SA West BS Reed E Moazen B Azim T Dolan K . Substance Use and HIV among female sex workers and female prisoners: risk environments and implications for prevention, treatment, and policies. J Acquir Immune Defic Syndr. 2015;69(Suppl 2):S110-7. 10.1097/QAI.0000000000000624 25978477 PMC4493865

[14] Fuge TG Tsourtos G Miller ER . A systematic review and meta-analyses on initiation, adherence and outcomes of antiretroviral therapy in incarcerated people. PLoS One. 2020;15(5):e0233355. 10.1371/journal.pone.0233355 32421754 PMC7233580

[15] Ickowicz S Salleh NAM Fairbairn N Richardson L Small W Milloy MJ . criminal justice system involvement as a risk factor for detectable plasma hiv viral load in people who use illicit drugs: a longitudinal cohort study. AIDS Behav. 2019;23(9):2634-9. 10.1007/s10461-019-02547-z 31236749 PMC6773261

[16] Milloy MJ Kerr T Buxton J Rhodes T Guillemi S Hogg R Dose-response effect of incarceration events on nonadherence to HIV antiretroviral therapy among injection drug users. J Infect Dis. 2011;203(9):1215-21. 10.1093/infdis/jir032 21459814 PMC3069731

[17] Rich JD Beckwith CG Macmadu A Marshall BDL Brinkley-Rubinstein L Amon JJ Clinical care of incarcerated people with HIV, viral hepatitis, or tuberculosis. Lancet. 2016;388(10049):1103-14. 10.1016/S0140-6736(16)30379-8 27427452 PMC5504684

[18] Semaille C Le Strat Y Chiron E Chemlal K Valantin MA Serre P Prevalence of human immunodeficiency virus and hepatitis C virus among French prison inmates in 2010: a challenge for public health policy. Euro Surveill. 2013;18(28):20524. 10.2807/1560-7917.ES2013.18.28.20524 23870097

[19] Cunningham EB Hajarizadeh B Bretana NA Amin J Betz-Stablein B Dore GJ Ongoing incident hepatitis C virus infection among people with a history of injecting drug use in an Australian prison setting, 2005-2014: The HITS-p study. J Viral Hepat. 2017;24(9):733-41. 10.1111/jvh.12701 28256027

[20] Danis K Doherty L McCartney M McCarrol J Kennedy H . Hepatitis and HIV in Northern Ireland prisons: a cross-sectional study. Euro Surveill. 2007;12(1):3. 10.2807/esm.12.01.00674-en 27938649

[21] Milloy MJ Buxton J Wood E Li K Montaner JS Kerr T . Elevated HIV risk behaviour among recently incarcerated injection drug users in a Canadian setting: a longitudinal analysis. BMC Public Health. 2009;9(1):156. 10.1186/1471-2458-9-156 19473508 PMC2695456

[22] Werb D Kerr T Small W Li K Montaner J Wood E . HIV risks associated with incarceration among injection drug users: implications for prison-based public health strategies. J Public Health (Oxf). 2008;30(2):126-32. 10.1093/pubmed/fdn021 18387974

[23] Altice FL Azbel L Stone J Brooks-Pollock E Smyrnov P Dvoriak S The perfect storm: incarceration and the high-risk environment perpetuating transmission of HIV, hepatitis C virus, and tuberculosis in Eastern Europe and Central Asia. Lancet. 2016;388(10050):1228-48. 10.1016/S0140-6736(16)30856-X 27427455 PMC5087988

[24] Stone J Martin NK Hickman M Hutchinson SJ Aspinall E Taylor A Modelling the impact of incarceration and prison-based hepatitis C virus (HCV) treatment on HCV transmission among people who inject drugs in Scotland. Addiction. 2017;112(7):1302-14. 10.1111/add.13783 28257600 PMC5461206

[25] Treloar C McCredie L Lloyd AR . The Prison Economy of Needles and Syringes: What Opportunities Exist for Blood Borne Virus Risk Reduction When Prices Are so High? PLoS One. 2016;11(9):e0162399. 27611849 10.1371/journal.pone.0162399 PMC5017673

[26] Wood E Li K Small W Montaner JS Schechter MT Kerr T . Recent incarceration independently associated with syringe sharing by injection drug users. Public Health Rep. 2005;120(2):150-6. 10.1177/003335490512000208 15842116 PMC1497693

[27] Michel L Trouiller P Chollet A Molinier M Duchesne L Jauffret-Roustide M ANRS-Coquelicot Study Group . Self-reported injection practices among people who use drugs in French prisons: Public health implications (ANRS-Coquelicot survey 2011-2013). Drug Alcohol Rev. 2018;37(Suppl 1):S268-76. 10.1111/dar.12620 29105203

[28] Cepeda JA Niccolai LM Lyubimova A Kershaw T Levina O Heimer R . High-risk behaviors after release from incarceration among people who inject drugs in St. Petersburg, Russia. Drug Alcohol Depend. 2015;147:196-202. 10.1016/j.drugalcdep.2014.11.021 25496706 PMC4297682

[29] Binswanger IA Stern MF Deyo RA Heagerty PJ Cheadle A Elmore JG Release from prison--a high risk of death for former inmates. N Engl J Med. 2007;356(2):157-65. 10.1056/NEJMsa064115 17215533 PMC2836121

[30] Kariminia A Law MG Butler TG Corben SP Levy MH Kaldor JM Factors associated with mortality in a cohort of Australian prisoners. Eur J Epidemiol. 2007;22(7):417-28. 10.1007/s10654-007-9134-1 17668280

[31] Merrall EL Kariminia A Binswanger IA Hobbs MS Farrell M Marsden J Meta-analysis of drug-related deaths soon after release from prison. Addiction. 2010;105(9):1545-54. 10.1111/j.1360-0443.2010.02990.x 20579009 PMC2955973

[32] Borquez A Beletsky L Nosyk B Strathdee SA Madrazo A Abramovitz D The effect of public health-oriented drug law reform on HIV incidence in people who inject drugs in Tijuana, Mexico: an epidemic modelling study. Lancet Public Health. 2018;3(9):e429-37. 10.1016/S2468-2667(18)30097-5 30122559 PMC6211569

[33] Gassowski M Nielsen S Bannert N Bock CT Bremer V Ross RS History of detention and the risk of hepatitis C among people who inject drugs in Germany. Int J Infect Dis. 2019;81:100-6. 10.1016/j.ijid.2019.01.015 30658167

[34] Michel L Lions C Van Malderen S Schiltz J Vanderplasschen W Holm K Insufficient access to harm reduction measures in prisons in 5 countries (PRIDE Europe): a shared European public health concern. BMC Public Health. 2015;15(1):1093. 10.1186/s12889-015-2421-y 26507505 PMC4624386

[35] Stöver H Hariga F . Prison-based needle and syringe programmes (PNSP) – Still highly controversial after all these years. Drugs Educ Prev Policy. 2016;23(2):103-12. 10.3109/09687637.2016.1148117

[36] Kamarulzaman A Reid SE Schwitters A Wiessing L El-Bassel N Dolan K Prevention of transmission of HIV, hepatitis B virus, hepatitis C virus, and tuberculosis in prisoners. Lancet. 2016;388(10049):1115-26. 10.1016/S0140-6736(16)30769-3 27427456

[37] Tarján A, Horváth G, Stöver H. European mapping of harm reduction interventions in prisons – revised version: July 2019. Frankfurt: Frankfurt University of Applied Sciences; 2019. Available from: https://www.researchgate.net/publication/335022280_European_Mapping_of_harm_reduction_interventions_in_prisons_-_Revised_version_July_2019

[38] Wolfe D Carrieri MP Shepard D . Treatment and care for injecting drug users with HIV infection: a review of barriers and ways forward. Lancet. 2010;376(9738):355-66. 10.1016/S0140-6736(10)60832-X 20650513

[39] MacDonald M Williams J Kane D . Barriers to implementing throughcare for problematic drug users in European prisons. Int J Prison Health. 2012;8(2):68-84. 10.1108/17449201211277192 25758018

[40] Stöver H, Jamin D, Sys O, Vanderplasschen W, Jauffret-Roustide M, Michel L, et al. Continuity of care for drug users in prisons and beyond in four European countries - Final report. Frankfurt: Frankfurt University of Applied Sciences; 2019. Available from: https://www.frankfurt-university.de/fileadmin/standard/Hochschule/Fachbereich_4/Forschung/ISFF/Forschungsprojekte/Abgeschlossene_Projekte/48h_out_finalreport_WP3_march2019.pdf

[41] Friedman SR Cooper HL Tempalski B Keem M Friedman R Flom PL Relationships of deterrence and law enforcement to drug-related harms among drug injectors in US metropolitan areas. AIDS. 2006;20(1):93-9. 10.1097/01.aids.0000196176.65551.a3 16327324

[42] Strathdee SA Hallett TB Bobrova N Rhodes T Booth R Abdool R HIV and risk environment for injecting drug users: the past, present, and future. Lancet. 2010;376(9737):268-84. 10.1016/S0140-6736(10)60743-X 20650523 PMC6464374

[43] Csete J Kamarulzaman A Kazatchkine M Altice F Balicki M Buxton J Public health and international drug policy. Lancet. 2016;387(10026):1427-80. 10.1016/S0140-6736(16)00619-X 27021149 PMC5042332

[44] Strathdee SA Beletsky L Kerr T . HIV, drugs and the legal environment. Int J Drug Policy. 2015;26(Suppl 1):S27-32. 10.1016/j.drugpo.2014.09.001 25265900 PMC4346482

[45] Hurley R . How to save drug users’ lives. BMJ. 2019;366:l5050. 10.1136/bmj.l5050 21868460

[46] Kruithof K, Davies M, Disley E, Strang L, Ito K. Study on alternatives to coercive sanctions as response to drug law offences and drug-related crimes. Brussels: European Commission; 2016. Available from: http://sisco.copolad.eu/web/uploads/documentos/PB_DGHOME_Publicacion_alternativas_encarcelamiento.pdf

[47] Gjersing L. Decriminalisation and possible impact on prison population and drug related problems. Session on Prison and drugs in Europe. Lisbon Addiction conference 2019; 23-25 October 2019, Lisbon, Portugal. Available from: https://www.lisbonaddictions.eu/lisbon-addictions-2019/presentations/decriminalisation-and-possible-impact-prison-population-and-drug-related-problems

[48] Hughes CE, Stevens A. The effects of the decriminalization of drug use in Portugal. Discussion paper. Oxford: The Beckley Foundation; 2007. Available from: https://kar.kent.ac.uk/13325/1/BFDPP_BP_14_EffectsOfDecriminalisation_EN.pdf.pdf

[49] Gonçalves R Lourenço A Silva SN . A social cost perspective in the wake of the Portuguese strategy for the fight against drugs. Int J Drug Policy. 2015;26(2):199-209. 10.1016/j.drugpo.2014.08.017 25265899

[50] Tamhane AR Westfall AO Burkholder GA Cutter GR . Prevalence odds ratio versus prevalence ratio: choice comes with consequences. Stat Med. 2016;35(30):5730-5. 10.1002/sim.7059 27460748 PMC5135596

[51] United Nations Statistics Division. Methodology - Standard country or area codes for statistical use (M49). New York: United Nations. [Accessed: 15 Aug 2020]. Available from: https://unstats.un.org/unsd/methodology/m49

[52] Institute for Crime & Justice Policy Research (ICPR). World prison brief. London: ICPR; 2020 [Accessed: 15 Aug 2020]. Available from: https://prisonstudies.org/world-prison-brief-data

[53] Dolan K Kite B Black E Aceijas C Stimson GV Reference Group on HIV/AIDS Prevention and Care among Injecting Drug Users in Developing and Transitional Countries . HIV in prison in low-income and middle-income countries. Lancet Infect Dis. 2007;7(1):32-41. 10.1016/S1473-3099(06)70685-5 17182342

[54] European Monitoring Centre for Drugs and Drug Addiction (EMCDDA). Lithuania country drug report 2019. Lisbon: EMCDDA; 2019. Available from: https://www.emcdda.europa.eu/system/files/publications/11341/lithuania-cdr-2019_0.pdf

[55] Kivimets K Uusküla A Lazarus JV Ott K . Hepatitis C seropositivity among newly incarcerated prisoners in Estonia: data analysis of electronic health records from 2014 to 2015. BMC Infect Dis. 2018;18(1):339. 10.1186/s12879-018-3242-2 30031373 PMC6054745

[56] Niculescu I Paraschiv S Paraskevis D Abagiu A Batan I Banica L Recent HIV-1 outbreak among intravenous drug users in Romania: evidence for cocirculation of CRF14_BG and subtype F1 strains. AIDS Res Hum Retroviruses. 2015;31(5):488-95. 10.1089/aid.2014.0189 25369079 PMC4426324

[57] McAuley A Palmateer NE Goldberg DJ Trayner KMA Shepherd SJ Gunson RN Re-emergence of HIV related to injecting drug use despite a comprehensive harm reduction environment: a cross-sectional analysis. Lancet HIV. 2019;6(5):e315-24. 10.1016/S2352-3018(19)30036-0 30981674

[58] Hagan H Pouget ER Des Jarlais DC Lelutiu-Weinberger C . Meta-regression of hepatitis C virus infection in relation to time since onset of illicit drug injection: the influence of time and place. Am J Epidemiol. 2008;168(10):1099-109. 10.1093/aje/kwn237 18849303 PMC2727245

[59] Maher L Chant K Jalaludin B Sargent P . Risk behaviors and antibody hepatitis B and C prevalence among injecting drug users in south-western Sydney, Australia. J Gastroenterol Hepatol. 2004;19(10):1114-20. 10.1111/j.1440-1746.2004.03438.x 15377287

[60] Tyndall MW Currie S Spittal P Li K Wood E O’Shaughnessy MV Intensive injection cocaine use as the primary risk factor in the Vancouver HIV-1 epidemic. AIDS. 2003;17(6):887-93. 10.1097/00002030-200304110-00014 12660536

[61] Allen EJ Palmateer NE Hutchinson SJ Cameron S Goldberg DJ Taylor A . Association between harm reduction intervention uptake and recent hepatitis C infection among people who inject drugs attending sites that provide sterile injecting equipment in Scotland. Int J Drug Policy. 2012;23(5):346-52. 10.1016/j.drugpo.2012.07.006 22940142

[62] Sypsa V Psichogiou M Paraskevis D Nikolopoulos G Tsiara C Paraskeva D Rapid decline in hiv incidence among persons who inject drugs during a fast-track combination prevention program after an hiv outbreak in Athens. J Infect Dis. 2017;215(10):1496-505. 28407106 10.1093/infdis/jix100 PMC5853582

[63] Cullen KJ Hope VD Croxford S Shute J Ncube F Parry JV . Factors associated with recently acquired hepatitis C virus infection in people who inject drugs in England, Wales and Northern Ireland: new findings from an unlinked anonymous monitoring survey. Epidemiol Infect. 2015;143(7):1398-407. 10.1017/S0950268814002040 25119383 PMC9507190

[64] Memedovic S, Iversen J, Geddes L, Maher L. Australian NSP Survey. National Data Report 2012-2016. Prevalence of HIV, HCV and injecting and sexual behaviour among NSP attendees. National Data Report 2012-2016. Sydney: Kirby Institute; 2017. Available from: https://kirby.unsw.edu.au/sites/default/files/kirby/report/ANSPS_National-Data-Report-2012-2016.pdf

[65] Fotiou A Kanavou E Antaraki A Richardson C Terzidou M Kokkevi A Drug Related Infectious Diseases (DRID) Medical Doctors Group of OKANA . HCV/HIV coinfection among people who inject drugs and enter opioid substitution treatment in Greece: prevalence and correlates. Hepatol Med Policy. 2016;1(1):9. 10.1186/s41124-016-0017-5 30288313 PMC5918725

[66] Hatzakis A Sypsa V Paraskevis D Nikolopoulos G Tsiara C Micha K Design and baseline findings of a large-scale rapid response to an HIV outbreak in people who inject drugs in Athens, Greece: the ARISTOTLE programme. Addiction. 2015;110(9):1453-67. 10.1111/add.12999 26032121 PMC4854521

[67] Folch C Casabona J Espelt A Majó X Meroño M Gonzalez V High Prevalence and Incidence of HIV and HCV Among New Injecting Drug Users With a Large Proportion of Migrants--Is Prevention Failing? Subst Use Misuse. 2016;51(2):250-60. 10.3109/10826084.2015.1092991 26820260

[68] Christensen PB Krarup HB Niesters HG Norder H Georgsen J . Prevalence and incidence of bloodborne viral infections among Danish prisoners. Eur J Epidemiol. 2000;16(11):1043-9. 10.1023/a:1010833917242 11421474

[69] Wiessing L. Epidemiology of HIV and viral hepatitis among people who inject drugs in Europe. Doctoral dissertation. Porto: University of Porto; 2017. Available from: https://repositorio-aberto.up.pt/bitstream/10216/110774/2/252084.docx

[70] Aebi MF, Tiago MM. Prisons and prisoners in Europe 2019: key findings of the SPACE I report. Strasbourg, Lausanne: Council of Europe, Universite de Lausanne; 2020. Available from: http://wp.unil.ch/space/files/2020/04/Key-Findings-2019_200406.pdf

[71] Tresó B Barcsay E Tarján A Horváth G Dencs A Hettmann A Prevalence and correlates of HCV, HVB, and HIV infection among prison inmates and staff, Hungary. J Urban Health. 2012;89(1):108-16. 10.1007/s11524-011-9626-x 22143408 PMC3284587

[72] Unlu A, Tammi T, Hakkarainen P. Drug decriminalization policy literature review: models, implementation and outcomes. Report number: 9/2020. Helsinki: National Institute for Health and Welfare; 2020. Available from: https://www.researchgate.net/publication/342131913_Drug_Decriminalization_Policy_Literature_Review_Models_Implementation_and_Outcomes

[73] Stevens A Hughes CE Hulme S Cassidy R . Depenalization, diversion and decriminalization: A realist review and programme theory of alternatives to criminalization for simple drug possession. Eur J Criminol. 2019;0(0):1477370819887514. 10.1177/1477370819887514

[74] Akiyama MJ Spaulding AC Rich JD . Flattening the curve for incarcerated populations - Covid-19 in jails and prisons. N Engl J Med. 2020;382(22):2075-7. 10.1056/NEJMp2005687 32240582 PMC7398586

[75] Simpson PL Butler TG . Covid-19, prison crowding, and release policies. BMJ. 2020;369:m1551. 10.1136/bmj.m1551 32312733

[76] Aebi MF, Tiago MM. Prisons and prisoners in Europe in pandemic times: an evaluation of the short-term impact of the COVID-19 on prison populations. Strasbourg, Lausanne: Council of Europe, Universite de Lausanne; 2020. Available from: http://wp.unil.ch/space/files/2020/06/Prisons-and-the-COVID-19_200617_FINAL.pdf

[77] Chaudry RV . The Precautionary Principle, public health, and public health nursing. Public Health Nurs. 2008;25(3):261-8. 10.1111/j.1525-1446.2008.00703.x 18477377

[78] Fischer AJ Ghelardi G . The precautionary principle, evidence-based medicine, and decision theory in public health evaluation. Front Public Health. 2016;4:107. 10.3389/fpubh.2016.00107 27458575 PMC4935673

[79] Wilson DP Donald B Shattock AJ Wilson D Fraser-Hurt N . The cost-effectiveness of harm reduction. Int J Drug Policy. 2015;26(Suppl 1):S5-11. 10.1016/j.drugpo.2014.11.007 25727260

