# Supplementary material for: Univariable associations between a history of incarceration and HIV and HCV prevalence among people who inject drugs across 17 countries in Europe 2006 to 2020 – is the precautionary principle applicable?
Source: Euro Surveill. 2021 Dec 9;26(49):2002093. doi: 10.2807/1560-7917.ES.2021.26.49.2002093 (PMC8662800; doi:10.2807/1560-7917.ES.2021.26.49.2002093)
Supplement: Supplement [file 20-02093_WIESSING_Supplement.pdf]

Supplementary Tables S1 and S2 (Wiessing et al., Eurosurveillance 2021)

This supplementary material is hosted by Eurosurveillance as supporting information alongside the article [Univariable associations between a history of incarceration and HIV and HCV prevalence among people who inject drugs across 17 countries in Europe – is the precautionary principle applicable?], on behalf of the authors, who remain responsible for the accuracy and appropriateness of the content. The same standards for ethics, copyright, attributions and permissions as for the article apply. Supplements are not edited by Eurosurveillance and the journal is not responsible for the maintenance of any links or email addresses provided therein.

Table S1. Study characteristics of the HIV testing data

**Country**	**Source /Reference**	**Year**	**Coverage**	**Sample size**	**Definition of Injectors**	**Study Design**	**Settings**
Austria	Inpatient Treatment Centre Lukasfeld (unpublished data)	2006-15	Vorarlberg	608	Ever	DT	DTC
Belgium	Belgium National Focal Point (unpublished data)	2008-11	Walloon region	363	Ever	SR	DTC
Croatia	S1; Croatian Institute for Public Health (unpublished data)	2007	Rijeka, Split, Zagreb	397	Ever	SP	DTC, NSP, LTS, PHL, HTC
Cyprus	Cyprus National Focal Point (unpublished data)	2006-15	National	888	Ever	DT	DTC
Estonia	Estonian National Focal Point, National Institute for Health Development (unpublished data)	2012-14	Kohtla-jarve, Tallin, Narva	1,277	Current	SP	NSP
France	S2,S3	2011	5 cities in France	898	Ever	SP-UAT	DTC, NSP, LTS
Germany	S4,S5	2011-14	8 cities in Germany	2,069	Current	SP	LTS
Greece	Greek National Focal Point for Drugs (unpublished data)	2006-15	Attica	934	Ever	DT	DTC
Hungary	S6	2014-15	National	1,180	Ever	SP	DTC, NSP, LTS
Latvia	S7	2007-14	Multiple cities & National	3,047	Current	SR/SP	NSP, STR
Lithuania	S8	2012-2014	Vilnius, Alytus, Visaginas, Klaipeda	530	Current	SP-UAT	NSP

Poland	S9	2009	Gdansk, Cracow	181	Ever	SP	LTS
Portugal	Multidisciplinary Information System – SIM (unpublished data)	2010-15	National	1,901	Ever	DT	DTC
Romania	National Anti-Drug Agency (unpublished data), available at http://ana.gov.ro/wp-content/uploads/2018/11/RN_2016.pdf)	2015	Bucharest	522	Ever	SP	STR
Sweden	InfCare NSP, National Quality Register	2013-20	National	8,512	Current	DT	LTS
UK	S10	2006-15	England & Wales	29,061	Ever	SP-UAT	DTC, NSP, LTS, OTH

Note: DT = Diagnostic testing; DTC = Drug treatment centres; HTC = HIV testing centres; LTS = Low-threshold services; NSP = Needle and syringe programmes; OTH = other settings; PHL = Public health laboratories; SP = Seroprevalence study; SP-UAT = Seroprevalence study with unlinked anonymous testing; SR = Self-reported test results; STR = Street.

Table S2. Study characteristics of the HCV testing data.

**Country**	**Source /Reference**	**Year**	**Coverage**	**Sample size**	**Definition of Injectors**	**Study Design**	**Settings**
Austria	Inpatient Treatment Centre Lukasfeld (unpublished data)	2006-15	Vorarlberg	608	Ever	DT	DTC
Croatia	S1; Croatian Institute for Public Health (unpublished data)	2007	Rijeka, Split, Zagreb,	397	Ever	SP	DTC, NSP, LTS, PHL, HTC
Cyprus	Cyprus National Focal Point (unpublished data)	2006-15	National	894	Ever	DT	DTC
Estonia	Estonian National Focal Point, National Institute for Health Development (unpublished data)	2013-14	Tallin, Narva	675	Current	SP	NSP
France	S2,S3	2011	5 cities in France	898	Ever	SP-UAT	DTC, NSP, LTS
Germany	S4,S5	2011-14	8 cities in Germany	2,071	Current	SP	LTS
Greece	Greek National Focal Point for Drugs (unpublished data)	2006-15	Attica	583	Ever	DT	DTC
Hungary	S6	2014-15	National	1,124	Ever	SP	DTC, NSP, LTS
Latvia	S7	2015	National	383	Current	SP	NSP, STR
Lithuania	S8	2012-2014	Vilnius, Alytus, Visaginas, Klaipeda	200	Current	SP-UAT	NSP
Poland	S9	2009	Gdansk, Cracow	180	Ever	SP	LTS
Portugal	Multidisciplinary Information System – SIM (unpublished data)	2010-15	National	1,518	Ever	DT	DTC
Romania	National Anti-Drug Agency (unpublished data), available at http://ana.gov.ro/wp-content/uploads/2018/11/RN_2016.pdf)	2015	Bucharest	521	Ever	SP	STR
Sweden	InfCare NSP, National Quality Register	2013-20	National	8,512	Current	DT	LTS

Turkey	S11	2008	Gaziantep city	168	Current	DT	STR
UK	S10	2006-15	England & Wales	28,536	Ever	SP-UAT	DTC, NSP, LTS, OTH

Note: DT = Diagnostic testing; DTC = Drug treatment centres; HTC = HIV testing centres; LTS = Low-threshold services; NSP = Needle and syringe programmes; OTH = other settings; PHL = Public health laboratories; SP = Seroprevalence study; SP-UAT = Seroprevalence study with unlinked anonymous testing; SR = Self-reported test results; STR = Street.

References for Supplementary Tables S1 and S2

S1. Kolarić B, Stajduhar D, Gajnik D, Rukavina T, Wiessing L. Seroprevalence of blood-borne infections and population sizes estimates in a population of injecting drug users in Croatia. Cent Eur J Public Health. 2010;18(2):104-9. http://dx.doi.org/10.21101/cejph.a3576 PMID:20939261

S2. Jauffret Roustide M, Pillonel J, Semaille C, et al. Estimation de la séroprévalence du VIH et de l'hépatite C chez les usagers de drogues en France. Premiers résultats de l'enquête ANRS-Coquelicot 2011. Bulletin épidémiologique hebdomadaire. 2013(39-40):504-9.

S3. Weill-Barillet L, Pillonel J, Semaille C, Léon L, Le Strat Y, Pascal X, et al. Hepatitis C virus and HIV seroprevalences, sociodemographic characteristics, behaviors and access to syringes among drug users, a comparison of geographical areas in France, ANRS-Coquelicot 2011 survey. Rev Epidemiol Sante Publique. 2016;64(4):301-12. http://dx.doi.org/10.1016/j.respe.2015.10.003 PMID:26904917

S4. Zimmermann R. DRUCK-Studie - Drogen und chronische Infektionskrankheiten in Deutschland. Ergebnisse der Pilotierung eines Sero- und Verhaltenssurveys bei i.v. Drogengebrauchern. 2012 2012. Available from: https://edoc.rki.de/handle/176904/175.

S5. Zimmermann R, Marcus U, Schäffer D, Leicht A, Wenz B, Nielsen S, et al. A multicentre sero-behavioural survey for hepatitis B and C, HIV and HTLV among people who inject drugs in Germany using respondent driven sampling. BMC Public Health. 2014;14(1):845. http://dx.doi.org/10.1186/1471-2458-14-845 PMID:25124485

S6. Dudás M, Rusvai E, Győri Z, et al. Official report on prevalence of infections related to injecting drug use in 2015. Budapest: National Centre for Epidemiology; 2015.

S7. Ķīvīte A, Vanaga D, Šibalova A, Skripste M, Zīle-Veisberga A, Kaupe R. Narkotiku lietošanas paradumi un tendences Latvijā. Narkotiku lietotāju kohortas pētījuma 10.posma rezultāti. Rīga: Slimību profilakses un kontroles centrs. 2017. Available from: https://www.spkc.gov.lv/lv/petijumi/narkotiku-lietosanas-paradumi-un-tendences-latvija-2017.pdf.

S8. Stonienė L, Narkauskaitė L, Davidavičienė E. ŽIV ir tuberkuliozės tarp švirkščiamųjų narkotikų vartotojų paplitimas, rizikos veiksniai ir paslaugų prieinamumas. [Prevalance of infections related with the use of narcotic and psychotropic substances among intravenous drug users] (in Lithuanian). Visuomenės sveikata. 2013;62:41-8. http://hi.simplit.lt/uploads/pdf/visuomenes%20sveikata/2013.3(62)/VS%202013%203(62)%20ORIG%20S%20ZIV%20ir%20tuberkulioze.pdf.

S9. Rosińska M. Oszacowanie występowania chorób zakaźnych (wirusowe zapalenie wątroby typu C i B, HIV) wśród osób przyjmujących środki odurzające we wstrzyknięciach w Gdańsku i w Krakowie [Estimation of the incidence of infectious diseases (hepatitis C and B, HIV) among people who inject drugs in Gdańsk and Kraków]. Final study report. (in Polish). Przeciwdziałania Narkomanii oraz Państwowy Zakład Higieny, raport z badania dostępny w siedzibie KBPN. 2009.

S10. Public Health England, Health Protection Scotland, Public Health Wales, Public Health Agency Northern Ireland. Shooting Up: Infections among people who injected drugs in the UK, 2015. An update, November 2016. London; 2016 11/2016. Available from: https://webarchive.nationalarchives.gov.uk/20170804142241/https://www.gov.uk/government/uploads/system/uploads/attachment_data/file/567231/Shooting_Up_2016_Update.pdf.

S11. Altan P. A Prevalance and Behavioral Study on HIV, Hepatitis B, Hepatitis C and Tuberculosis among Intravenous Drug Users in Gaziantep 2009. Ankara: Turkish Ministry of Health; 2009. Available from: http://www.emcdda.europa.eu/drugs-library/prevalance-and-behavioral-study-hiv-hepatitis-b-hepatitis-c-and-tuberculosis-among-intravenous-drug-users-gaziantep-2009_en
